# A Systematic Review of the Mechanisms Underlying Treatment of Gastric Precancerous Lesions by Traditional Chinese Medicine

**DOI:** 10.1155/2020/9154738

**Published:** 2020-04-26

**Authors:** Liangjun Yang, Jiali Li, Zhipeng Hu, Xiangzhen Fan, Tiantian Cai, Hengli Zhou, Huafeng Pan

**Affiliations:** ^1^Department of Gastroenterology, Tongde Hospital of Zhejiang Province, Hangzhou 310012, China; ^2^Guangzhou University of Chinese Medicine, Guangzhou 510405, China; ^3^The Hospital of Chengdu University of Traditional Chinese Medicine, Chengdu 610075, China; ^4^The Second Affiliated Hospital of Guangzhou University of Chinese Medicine, Guangzhou 510000, China

## Abstract

Gastric precancerous lesions (GPLs) are an essential precursor in the occurrence and development of gastric cancer, known to be one of the most common and lethal cancers worldwide. Traditional Chinese medicine (TCM) has a positive prospect for the prevention and therapy of GPL owing to several advantages including a definite curative effect, fewer side effects compared to other treatments, multiple components, and holistic regulation. Despite these characteristic advantages, the mechanisms of TCM in treating GPL have not been fully elucidated. In this review, we summarize the current knowledge with respect to herbal formulations and the therapeutic mechanisms of TCM active ingredients for GPL. This paper elaborates on the mechanisms of TCM underlying the prevention and treatment of GPL, specifically those that are linked to anti-*H. pylori*, anti-inflammation, antiproliferation, proapoptotic, antioxidation, antiglycolytic, and antiangiogenesis effects.

## 1. Introduction

Gastric cancer is known to involve one of the most common and aggressive tumors in the digestive system. According to a report by the Global Cancer Observatory (GCO), gastric cancer is the fifth most frequently diagnosed cancer and ranks as the third leading cause of cancer-related deaths, with a 5-year overall survival rate of less than 25% [1, 2]. It remains the most prevalent cancer in Eastern Asia, including China, Japan, and Korea [3]. Although the incidence rate of gastric cancer has been decreasing for several decades owing to the changing of unhealthy behaviour, the improvement in disease awareness, and the elimination of *H*. *pylori* [4–6], the incidence and mortality rate of gastric cancer was 679.1 and 498.0 per 100 000, respectively [7]. Gastric cancer is still identified as the leading cause of cancer deaths in China [8].

It is well known that the occurrence of gastric cancer is a continuous multistage biological process starting with chronic superficial gastritis, atrophic gastritis, intestinal metaplasia, and finally dysplasia and adenocarcinoma [9]. Gastric precancerous lesions (GPLs), which include intestinal metaplasia and dysplasia [10], are inevitable in the occurrence of gastric cancer. Evidence has shown that the annual incidence of gastric cancer was 0.25% for intestinal metaplasia, 0.6% for mild-to-moderate dysplasia, and 6% for severe dysplasia within 5 years after diagnosis [11]. Based on a multicenter national study in China, intestinal metaplasia and dysplasia accounted for 23.6% and 7.3% of patients with gastritis, respectively [12]. Thus, effective treatment of GPL in China is imperative to prevent progression to gastric cancer.

**Figure 1:**
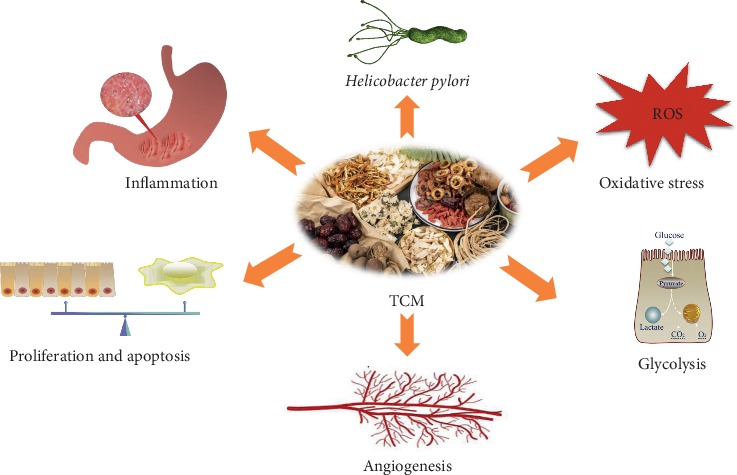
Potential mechanisms of traditional Chinese medicine for GPL therapy.

At present, *Helicobacter pylori* (*H. pylori*) elimination and endoscopic mucosal resection are clinically conducted in the Western medicine system for patients with GPL. While endoscopic mucosal resection is only used to treat severe gastric dysplasia [13], few definite guidelines are available regarding the management of low-grade dysplasia [14], which greatly restricts the treatment of GPL. Therefore, seeking complementary and alternative treatments for GPL is highly significant. Traditional Chinese medicine (TCM) is a comprehensive medical system that studies human physiology, pathology, disease diagnosis, and control of such disciplines. It has been widely used in the treatment and prevention of various diseases since 200 AD and has played a vital role in improving the health of Asian people, especially in China [15]. The use of herbal medicine within this system has been viewed as an effective measure to prevent chronic diseases. It has been proven that the use of TCM is effective in blocking and reversing majority of GPL developments [16–18]. As shown in Figure 1, this paper elucidates forms of TCM treatment for the prevention and treatment of GPL, including herbal formulations and TCM active ingredients. Underlying therapeutic mechanisms are also described.

## 2. Clinical Effects of TCMs on GPL

TCM has been applied to the prevention and treatment of digestive system problems in China for thousands of years. Clinical manifestations of chronic gastritis have been described in a Chinese Canon of Internal Medicine called *Huangdi Neijing,* between 770 and 221 BC. An example of this is “*Pi Man*,” described in the document as gastric distention and fullness. Ancient doctors have also developed treatment methods for such diseases, which are recorded in ancient classical texts such as the *Shang Han Lun*, one of the earliest books on TCM clinical practice dating back to somewhere between 150 and 219 AD. Included in this text is a prescription named “*Ban-Xia-Xie-Xin-Tang*” for the treatment of *Pi Man*. Significant evidence has shown that *Ban-Xia-Xie-Xin-Tang* influences anti-inflammatory activity and can be utilized to treat various digestive diseases such as gastritis, functional dyspepsia, and peptic ulcer [19–21]. These findings indicate the utility of TCM in the prevention and treatment of digestive diseases.

With the promotion and application of evidence-based medicine in the field of TCM, an increasing number of studies have shown that Chinese medicine can be effective in the treatment of GPL. Based on the characteristics of TCM-defined syndromes of chronic atrophy gastritis, qi deficiency, qi stagnation, blood stasis, phlegm turbidity, heat, and yang deficiency were considered to be the core pathogenetic factors [22, 23]. Since the spleen is responsible for the origination of qi, blood, and fluid and transporting them throughout the body to support the body's overall function, its dysfunction always results in the deficiency and stagnation of qi, blood, and fluid. Therefore, strengthening the spleen, which promotes the formation and transportation of nutrients, as well as the restoration of gastric, should be incorporated into the key treatment of GPL. Five TCM medicines have been empirically reviewed within existing literature, as outlined in Table 1. *Sijunzi decoction*, a traditional Chinese herbal formula, has been widely used to treat gastrointestinal disorders. Several meta-analyses demonstrated that *Sijunzi decoction* was efficacious and safe for atrophy chronic gastritis and gastric cancer [24, 30]. Clinical studies have shown that *Sijunzi decoction* or modified *Sijunzi decoction* can reverse pathological gastric changes (including chronic atrophic gastritis, intestinal metaplasia, and gastric epithelial dysplasia) and is effective in treating patients with fatigue and tiredness [24–26, 31]. *Weiansan,* a classical formula commonly used in the treatment of GPL, was found to have definite effects, including suppression of glandular atrophy, intestinal metaplasia, and dysplasia and elimination of *H. pylori* [17]. Another example is *Moluodan,* a Chinese-patent medicine generally applied for treating CAG and GPL in clinical practice. In a multicentered, double-blind, randomized controlled trial, histological improvements were noted with gastric dysplasia, and the medicine was found to be significantly more effective in improving epigastric pain, epigastric suffocation, belching, and appetite compared to folic acid [27]. Similarly, *Weikangfu granules* are a classic prescription for the treatment of chronic gastritis in spleen-deficiency syndrome. Patients who suffered from atrophy gastritis with intestinal metaplasia or atypical hyperplasia were treated using *Weikangfu granules* and showed significant improvement in symptoms [28]. Finally, the *Weixibaonizhuan pill,* a Chinese herbal compound formula, was designed and utilized for treating chronic atrophic gastritis with intestinal metaplasia and dysplasia. The therapeutic effects of these pills among 30 patients with GPL following three months of treatment were significantly higher in mild and moderate cases than those with severe symptoms [29]. These results therefore indicated that *Weixibaonizhuan pills* could be particularly beneficial during the early stages of GPL when symptoms are mild or moderate. Taken together, it can be seen that Chinese medicine has a definitive therapeutic effect in GPL, which could not only improve symptoms and reduce discomfort but can also reverse pathological processes.

## 3. Therapeutic Mechanisms of TCM in GPL

The therapeutic mechanisms of TCM in treating GPL have been investigated in numerous studies. TCMs have been shown to have anti-*H. pylori*, anti-inflammation, antiproliferation, proapoptotic, antioxidation, antiangiogenesis, and metabolism regulatory effects, which are summarized in Table 2.

### 3.1. *Helicobacter pylori* Elimination

*H. pylori, which* specifically colonizes the surface of the gastric mucosal epithelium in the antrum of the stomach, is involved in various gastric malignant diseases. According to epidemiological studies, *H. pylori* infects more than 50% of the population in Asia, south and east Europe, and South America [52] and has resulted in a large incidence of gastric cancers [53]. Following infection, *H. pylori* can produce a series of toxins, leading to the development of gastric cancer. CagA and VacA, secreted by *H. pylori*, have several cytotoxic effects, including stimulating cell proliferation, reducing epithelial cell apoptosis, activating inflammatory signals, producing reactive oxygen species, and finally inducing accumulation of multiple genetic variants, all of which increase the risk of gastric carcinoma [54, 55]. It is also well known that *H. pylori* infection is associated with the development of precancerous lesions such as atrophic gastritis, gastric intestinal metaplasia, and gastric dysplasia [56]. Therefore, eliminating *H. pylori* is an effective clinical protocol to prevent the progression of gastric pathology.

There has been some research regarding the use of TCM for the elimination of *H. pylori*. *Weiansan*, a Chinese medicine formula composed of many ingredients including *astragalus root*, *pilose asiabell root*, *red sage root*, *bighead atractylodes rhizome*, *scorpion*, and *wheat*, functions to promote blood flow, invigorate the spleen, and replenish qi. Clinical studies have shown that the total effect rate of *Weiansan* in the treatment of GPL is about 85.70% and that the medicine can improve symptoms by reversing pathological damage and eliminating *H. pylori* [17]. Wei Bi Mei granules, consisting of five Chinese herbs including *licorice*, *cortex frangulae*, *fructus foeniculi*, *aloe*, and *Acorus gramineus*, have been used to treat several digestive diseases. Li et al. [32, 57] conducted an in vivo experiment to assess the efficacy and safety of *Wei Bi Mei* in the elimination of *H. pylori.* The results showed that *Wei Bi Mei* granules is effective and safe in the treatment of *H. pylori* infection, and further evaluation regarding its anti-*H. pylori* activity is warranted. Tinospora sagittata (Oliv.) Gagnep. var. craveniana (S. Y. Hu) Lo (TSG) is a traditional Chinese herb that has been used to treat infectious diseases owing to its antibacterial, anti-inflammatory, and antinociceptive activities [58]. To investigate the anti-*H. pylori* effect of TSG, *H. pylori* SCYA201401 (isolated from pigs) and *H. pylori SS1* were used for in vitro and murine experiments. Rong et al. [33] concluded that TSG and its major component, palmatine, demonstrated bactericidal activity against *H. pylori* in both settings, which was similar to the antibacterial effect of clarithromycin. *Jinghua Weikang* capsule is a proprietary Chinese medicine used for chronic gastritis and peptic ulcer. It mainly consists of *Chenopodium ambrosioides* L. and *Adina pilulifera* (Lam.) Franch., belonging to the Chenopodiaceae family [59, 60]. Liu et al. [34] investigated the bactericidal activity of this capsule and its individual herb *Chenopodium ambrosioides* L. on drug-resistant *H. pylori* strains obtained from gastric biopsies of clinical patients. Both the *Jinghua Weikang* capsule and *Chenopodium ambrosioides* L. displayed effective germicidal action against antibiotic-resistant *H. pylori*, which offered a novel approach to treat *H. pylori* infection. *Atractylodes lancea* volatile oils, containing *β*-caryophyllene, *β*-sesquiphellandrene, atractylone, and caryophyllene oxide extracted from *Atractylodes lancea,* have been reported to have antimicrobial function [61, 62]. Yu et al. [35] investigated the antibacterial effect of *Atractylodes lancea* volatile oils on the planktonic growth and biofilm formation of *H. pylori* cocultured with gastric epithelial cells. The study first discovered that volatile oils could reduce biofilm formation of *H. pylori* and could reduce the expression of Cag A and the inflammatory cytokine IL-8 secreted by GES-1 cells. This suggested that *Atractylodes lancea* volatile oils were a potential drug against *H. pylori* infection. Several Chinese medicine monomers can also play a role in *H. pylori* elimination. Flavonoid glycosides of *Polygonum capitatum,* patchouli alcohol, geniposide, and genipin were investigated for the treatment of gastritis caused by *H*. *pylori*. The results demonstrated that these monomers can reduce inflammatory damage by eliminating *H*. *pylori*, indicating that these compounds can be prepared as new drugs for *H. pylori* infection [36–38]. These observations not only indicated the direct anti-*H. pylori* activity of TCM but also highlighted its potential effects in the treatment of *H. pylori*-associated GPL.

Although the elimination of *H*. *pylori* infection is the primary treatment for GPL, this strategy has become increasingly difficult to sustain due to the emergence of antibiotic-resistant *H. pylori* strains [63]. Despite this, an increasing number of studies have found that TCM can play a role in the elimination of *H*. *pylori* and have attracted the attention of researchers worldwide.

### 3.2. Anti-Inflammation

Chronic gastric inflammation is one of the key factors contributing to the development of gastric carcinoma. Inflammatory mediators can induce cytogenetic and epigenetic changes that trigger imbalances in cell homeostasis and lead to alterations in critical pathways responsible for cancer development and progression [64]. As an important step in the pathogenesis of the stomach, precancerous lesions caused by various infectious and noninfectious agents are closely related to the deleterious effects of inflammation [65]. Celecoxib, a selective nonsteroidal anti-inflammatory drug used to inhibit cyclo-oxygenase-2 (COX-2), has been shown to have beneficial effects on the regression of advanced gastric lesions [66]. Thus, attenuation of gastric mucosal inflammation could be an effective preventive and therapeutic strategy in the treatment of GPL.

TCM drugs having anti-inflammatory properties include *Weiqi decoction*, which is an empirical formula consisting of *Radix Angelicae Sinensis*, *Radix Astragali*, *Radix Codonopsis*, *Rhizoma Curcumae*, *Fructus Aurantii*, *Fructus Akebiae*, and *Herba Taraxaci*. It is used for GPL treatment and fortifies the spleen, removes blood stasis, and harmonizes the stomach [67]. Experimental studies have shown that *Weiqi decoction* had an inhibitory effect on gastric inflammation by reducing COX-2 and increasing PGE2 in chronic atrophy gastritis rats with precancerous lesions [39]. Among the decoctions, herbs such as *Radix Angelicae Sinensis* [68], *Radix Astragali* [69], *Radix Codonopsis* [70], *and Rhizoma Curcumae* [71] were reported to exert anti-inflammatory effects by modulating the release of inflammatory mediators, which indicated that attenuating the inflammatory injury is a key mechanism of *Weiqi decoction* in the treatment of GPL. *Panax ginseng* is a herb used for more than 2000 years as a tonic to relieve fatigue [72]. Numerous studies conducted on *Panax ginseng* have identified pharmacological functions including anti-inflammation, antioxidative stress, and immunoregulation [73, 74]. Moreover, it was also found to protect the gastric epithelial cells by increasing the histological grade of neutrophil infiltration, intestinal metaplasia, and hyperplasia and by suppressing the expression of inflammatory mediators such as keratinocyte chemoattractant, IL-1*β*, and iNOS [40, 75]. As a feature of TCM, the use minerals for treatment have been documented in the *Shennong Bencao Jing* (about 200 BC–200 AD). *Muscovite*, a natural ore composed of an insoluble double silicate of aluminum and magnesium, is used in the management of gastric diseases in TCM. Studies have shown that muscovite powder promotes the secretion of gastric acid and gastric pepsin and could increase the number of chief and parietal cells as well as G and D cells to protect the gastric mucosa [41]. In addition, muscovite's ability to reverse gastric mucosal atrophy and intestinal metaplasia is inextricably linked to the reduction of inflammatory mediators including TNF-*α*, PGE2, and IL-1*β* [76].

Taken together, the protective effect of TCM against GPL is closely related to its anti-inflammatory activity. With further studies, an increasing number of anti-inflammatory mechanisms of herbs or extracts from TCM will be understood, thereby opening up some novel ways for further therapy of GPL genesis.

### 3.3. Regulating the Proliferation and Apoptosis of GPL

One of the most fundamental traits of cancer cells is its ability to sustain chronic proliferation [77]. In the progression from chronic gastritis to gastric cancer, abnormalities in the proliferation of gastric mucosal cells have been observed in gastric dysplasia [78]. Apoptosis is the programmed cell death that maintains cell survival and death balance to maintain the cell populations in tissues. It also acts as a defense mechanism when cells are destroyed by diseases, microorganisms, or noxious agents [79]. Abnormal apoptosis is associated with the formation of gastrointestinal malignancies [80] and has a significant impact during the formation of GPL [81]. The occurrence of GPL is closely related to the dynamic imbalance of cell proliferation and apoptosis. Therefore, modulating the proliferation and apoptosis of gastric mucosal epithelial cells is an important way to treat precancerous lesions.

*Xiao Tan He Wei* decoction is usually used to reverse precancerous lesions and is proven to be useful in relieving clinical symptoms of GPL [42]. This decoction contains five herbs, including *radix bupleuri*, *rhizome pinelliae*, *Poria cocos*, *Coptis chinensis*, and *radix glycyrrhizae preparata*. Across in vitro and in vivo experiments, Xu et al. [42] found that *Xiao Tan He Wei* decoction can inhibit cell proliferation and induce apoptosis of GPL cells by intervening in the NF-*κ*B pathway. Caspase-3 and Ki67 are considered apoptosis and proliferative cell markers in tumor formation, respectively [82, 83]. A recent study showed that the *Weiqi* decoction introduced above could enhance the protein expression of cleaved caspase-3 and decrease the protein expression of Ki67 in pyloric gastric mucosa in GPL rats, indicating that this decoction could regulate the imbalance of proliferation and apoptosis [39]. *Ginkgo biloba* is an ancient herb that has been used for thousands of years in China. In recent years, research has pointed towards its antitumor effects because of which it has been used to treat gastric cancer by inducing cell apoptosis and inhibiting cell proliferation [84, 85]. Jiang et al. [43] demonstrated that *Ginkgo biloba* can block GPL progression via the regulation of cell proliferation and apoptosis. *Hericium erinaceus*, an edible mushroom for mucosal protection, is widely used to treat gastritis, gastric ulcers, and gastric tumors. Wang et al. [44] purified polysaccharides from *Hericium erinaceus* and examined its effects on a gastric precancerous cell line modeled by N-methyl-N′-nitro-N-nitrosoguanidine. They discovered that polysaccharides could effectively induce cell apoptosis and cell cycle arrest at the G0/G1 phase in the precancerous cell line by regulating the expression of Bax, Bcl-2, and caspase-3. *Astragaloside IV* is a saponin extracted from *Astragalus membranaceus*, a traditional Chinese herb used to treat various diseases and physical disorders. Cai et al. [45] confirmed that *astragaloside IV* can improve gastric precancerous conditions by downregulating Bcl-XL and Bcl-2/Bax and upregulating caspase-3 to maintain the balance between cell proliferation and apoptosis.

In summary, the imbalance of cell proliferation and apoptosis is a critical feature of GPL, and Chinese medicine can exert a significant impact by regulating this process in gastric cancer prevention.

### 3.4. Inhibition of Oxidative Stress

The balance of redox homeostasis is the basis for cells to maintain their survival and maintain normal functions. Oxidative stress occurs when there is an imbalance between the production and removal of reactive oxygen/nitrogen species, which is one of the characteristics of tumor cells [86]. Studies have revealed that the reactive oxygen species (ROS) generated by cells under oxidative stress are an essential factor in the pathogenesis of gastrointestinal mucosal diseases [87]. ROS produced by the gastric immune and epithelial cells can damage the gastric mucosa and induce irreversible damage to proteins, organelle lipids, and DNA, which leads to GPL and ultimately to gastric cancer [88, 89]. Moreover, dietary supplementation with antioxidant micronutrients has been demonstrated to be efficient in increasing the rate of regression of cancer precursor lesions [90]. Thus, reducing the level of oxidative stress should be a reliable treatment for GPL.

Patients who suffered from gastric intestinal metaplasia and atypical hyperplasia were treated with *Weikangfu* granules, and changes in lipid peroxide and superoxide dismutase were observed before and after treatment. After therapy, the patient's TCM syndrome and gastric mucosal pathology were significantly improved, while lipid peroxide and superoxide dismutase were markedly decreased [28]. The *Jianpiyiwei* capsule is a compound Chinese medicine used to decrease the incidence of gastric dysplasia and intestinal metaplasia by replenishing qi, invigorating the spleen, and promoting blood flow. To clarify the mechanism of action, Shi et al. [46] explored the effect of *Jianpiyiwei* capsule on GPL rats by measuring the mucosal malonic dialdehyde, a metabolite of lipid peroxidants. After therapy, the malonic dialdehyde content of the gastric mucosa in the GPL rats decreased significantly, which implied that the *Jianpiyiwei* capsule provided protective action by regulating free radicals. Above all, it showed that Chinese herbal compounds have a protective effect on gastric mucous by reducing the level of oxidative stress. *Curcumin*, a compound extracted from the root of the turmeric plant, is a highly potent antitumor agent that has been shown to be active against gastrointestinal tumors, including esophageal cancer [91], gastric cancer [92], and colorectal cancer [93]. In addition, *curcumin* can reduce precancerous lesions and tumorigenesis in the glandular stomach and has a therapeutic effect against oxidative stress-mediated gastric diseases, indicating that this monomer has a chemopreventive effect on the occurrence of adenocarcinoma [47, 94]. The *Ginkgo biloba* extract described above has also been proven to treat GPL by regulating cell proliferation and apoptosis. Moreover, antioxidative stress is one of the methods used to treat intestinal metaplasia and dysplasia. Through the intervention of *Ginkgo biloba* extract on GPL rat, Jiang et al. [43] confirmed that the activity of superoxide dismutase was increased and the concentration of malondialdehyde was decreased, which suggested its protective effects against oxidative stress. *Dendrobium officinale* polysaccharides are herbal medicine extracts belonging to genus Dendrobium, which is regarded as a prized folk medicine. In the study of *Dendrobium officinale* polysaccharides, researchers found that the polysaccharides have protective effects against 1-methyl-3-nitro-1-nitrosoguanidine-induced precancerous lesions of gastric cancer by activating the NRF2 pathway and its related antioxidant enzymes, heme oxygenase-1, and NADPH quinone oxidoreductase-1 [48].

In summary, oxidative stress plays a vital role in the occurrence, development, and deterioration of GPL. Many Chinese medicines or their active constituents have been shown to elicit beneficial effects on antioxidant activity and cleaning free radicals. These findings show that block oxidative stress is an effective option for TCM in the treatment of GPL.

### 3.5. Inhibition of Glycolysis

Aerobic glycolysis or the Warburg effect is a metabolic phenotype characterized by energy production in an oxygen-independent manner. As one of the hallmarks of cancer, it ensures that tumor cells can fully and quickly acquire energy and biosynthetic intermediates from glucose, thus promoting tumor progression and resistance to chemotherapeutic agents [95, 96]. In recent years, studies have shown that reversing the glycolytic phenotype in tumor cells to oxidative phosphorylation can induce tumor cell death. Thus, intervention on tumor glycolysis can be a new strategy and therapeutic option for cancer therapy [97, 98]. As an antecedent of gastric adenocarcinoma, it has been found that chronic atrophic gastritis has abnormal energy metabolism, such as tricarboxylic acid cycle, glycolysis, membrane metabolism, and catabolism [99]. Moreover, emerging evidence has shown that aerobic glycolysis occurs at the stage of GPL, suggesting that altered glycolysis is a fundamental feature in the origin of gastric cancer [49].

Currently, some scholars have studied Chinese medicine to treat GPL by regulating glycolysis. *Astragalus membranaceus* Bunge has long been used to treat gastrointestinal diseases in China. As the main active ingredient of Astragalus, *astragaloside IV* has been shown to attenuate the severity of gastric mucosal damage, as evidenced by anti-inflammatory and antioxidative stress [100, 101]. It was also found that *astragaloside IV* can reverse MNNG‐induced GPL in rats by inhibiting glycolysis through the dual regulation of the p53/miRNA‐34a/LDHA and p53/TIGAR pathways. Meanwhile, it can also suppress the glycolic process by restoring the abnormal expression of MCT1/4, CD147, and HIF‐1α in GPL, which provides a new therapeutic strategy for the prevention of gastric cancer [49]. HIF-1*α*, an active subunit of HIF-1, which is essential for tumor cells to adapt to a low-oxygen environment, has a vital role in aerobic glycolysis and tumorigenesis [102]. Activated HIF-1*α* in tumor cells can increase the expression and activity of glycolytic enzymes, including GLUT1 and HK2, to accelerate the glycolytic rate [103]. Chinese medicine prescriptions such as *Weipixiao* formula and *Weiqi* decoction have been proven to attenuate GPL by suppressing levels of HIF-1*α* [16, 39], suggesting their potential antiglycolytic function.

In summary, there is significant evidence that using TCM that targets disordered glycolysis occurring in the precancerous period is effective in the treatment of GPL.

### 3.6. Inhibition of Angiogenesis

Angiogenesis is a physiological process in which new blood vessels grow from pre-existing vessels. As the fundamental for solid tumor growth and metastasis, angiogenesis provides oxygen and nutrients through blood vessels to the tumor site and results in hyperpermeable vasculature with a twisted appearance, both of which promote tumor progression [104, 105]. Studies have demonstrated that the positive expression of p53, iNOS, and VEGF, fundamental biomarkers of the angiogenic process, increased with lesion progression from chronic atrophic gastritis to intestinal metaplasia to dysplasia [106]. Not only is this a characteristic of invasive tumors but also an early event during GPL genesis [107, 108], which provides an emerging therapeutic target.

Based on these data, the antiangiogenesis function of TCM was investigated. *Weipixiao* is a complex TCM prescription consisting of 6 herbs, including *Astragalus membranaceus*, *Pseudostellaria heterophylla*, *Atractylodis macrocephala*, *Curcuma zedoaria*, *Salvia miltiorrhiza*, and *Hedyotis diffusa* Willd. Clinical studies have shown that *Weipixiao* could alleviate clinical symptoms and reduce precancerous lesions of gastric cancer [109, 110]. Another study indicated that its pharmacological mechanisms might be associated with modulation of the VEGF signaling pathway [111]. Experiments on rats with GPL verified that *Weipixiao* could attenuate early angiogenesis with a concomitant regression of precancerous lesions by suppressing VEGF activation [16]. The *Weining* granules, a Chinese prescription composed of *Radix Astragali*, *Poria cocos*, *Rhizoma Curcumae Longae,* and *Fructus Lycii*, has been applied in the integrative treatment of patients with GPL. Clinical studies have demonstrated that patients taking *Weining* granules appear to be more effective in improving the gastric precancerous state and the main symptoms, including gastric pain, distension, and anorexia, with a low risk of adverse events. The therapeutic mechanisms of Weining granules in GPL might be related to angiogenesis inhibition through the downregulation of VEGF and microvessel density [50]. *β*-Glucan, a polysaccharide found in cereal, seaweeds, and *Poria cocos*, has many effects on human health, including antitumor, anti-infection, blood cholesterol thinning, and immune-modulating properties [112, 113]. Studies on the antitumor effect of *β*-glucan indicated that *β*-glucan could promote immune cell accumulation in tumors, regulate cell apoptosis and proliferation, and inhibit angiogenesis by suppressing VEGF expression, leading to slow progression of tumors [114]. Based on this background, the mechanism of action of *β*-glucan on GPL was further studied. Desamero et al. [51] investigated *β*-glucan against the development of gastric dysplasia in A4gnt KO mice. After 3 weeks of treatment with *β*-glucan, the gastric dysplasia in mice was significantly relieved, which was associated with inhibition of cell proliferation and angiogenesis.

Taken together, these findings suggest that inhibition of angiogenesis is an important mechanism for the treatment of GPL by TCM.

## 4. Conclusions

As the penultimate stage in gastric carcinogenesis, early intervention and monitoring of GPL are critical in the prevention of gastric cancer. Although some spontaneous regression of mild and moderate or even high-grade gastric dysplasia has been observed [13, 115, 116], these may be partly attributed to sampling errors and differences in histological interpretation of dysplasia between observers [13, 117]. In addition, little is known about the possibilities of natural processes that change precancerous conditions [118]. Thus, early prevention of GPL is essential to reduce the incidence of gastric cancer. Currently, Western medicine treats GPL mainly by endoscopic therapy, *H. pylori* elimination, oral administration of COX inhibitors, nonsteroidal anti-inflammatory drugs, or antioxidant vitamins [119]. However, these are accompanied by side effects and risk of GPL recurrence after endoscopic treatment [120, 121]. Thus, it is imperative to develop safer and more effective options for GPL therapy. In recent years, TCM has become a research hotspot owing to its advantages of definite curative effect, few side effects, multiple components, and holistic regulation. The promise of Chinese herbs as effective therapies for GPL has been assessed by clinical evaluations and experimental studies. These results support the potential application of these TCMs as novel therapeutic agents to treat patients with GPL.

Although TCM is of great value in clinical practice and has been confirmed to be effective in curing GPL, the active ingredients in herbs, the potential targets, and the exact molecular mechanisms are still not completely understood. Thus, more research should be conducted in this regard. These efforts could include multicenter, double-blind, randomized, and controlled clinical trials to confirm the effectiveness and safety of TCMs; in vivo and in vitro experiments that involve isolation and screening of major bioactive components in herbs and evaluation of their molecular mechanisms of action; and exploration of new analytical tools (such as network pharmacology) to study the integrated mechanisms of TCM compounds and their interactions. More attention should be paid to research on TCM toxicity problems, and new analytical techniques and methods should be used to investigate the toxicity mechanisms of TCM. Well-designed clinical trials and experimental studies should be conducted to facilitate a better understanding of the mechanism of TCM and promote the modernization of TCM in the treatment of GPL.

## Figures and Tables

**Table 1:** Chinese herbal formulations for GPL.

Names	Origins	TCM function	Reference
Sijunzi decoction	*Radix Ginseng, Poria cocos, Rhizoma Atractylodis Macrocephalae, and Radix Glycyrrhizae*	Strengthens the spleen, replenishes qi	[24–26]

Weiansan	*Astragalus root, pilose asiabell root, bighead atractylodes rhizome, cinnamon bark, red sage root, scorpion, rhizome corydalis, fructus aurantii, processed grain, and wheat*	Strengthens the spleen, replenishes and regulates qi, invigorates blood	[17]

Moluodan	*Bulbus Lilii, Rhizoma Alismatis, Poria, Radix Notoginseng, Radix Sanguisorbae, Rhizoma Ligustici Chuanxiong, Rhizoma Acori Tatarinowii, Radix Ophiopogonis, Radix Linderae, Herba Artemisiae Scopariae, Radix Scrophulariae, Pollen Typhae, Radix Paeoniae Alba, Endothelium Corneum Gigeriae Galli, Herba Dendrobii, Radix Angelicae Sinensis, Rhizoma Corydalis*, *and Rhizoma Atractylodis Macrocephalae*	Regulates stomach, promotes flow of qi, strengthens the spleen, regulates qi, activates meridians	[27]

Weikangfu granule	*Radix Astragali, Radix Astragali seu Hedysari, Rhizoma Atractylodis Macrocephalae, Radix Glycyrrhizae, Rhizoma Corydalis, Rhizoma Coptidis, and Radix Paeoniae Alba*	Strengthens the spleen, replenishes qi	[28]

Weixibaonizhuan pill	*Radix Codonopsis pilosulae, Radix Salviae Miltiorrhizae, Herba Solani Nigri, Radix et Rhizoma Rhei, and Rhizoma coptidis*	Strengthens the spleen and stomach, consolidates the constitution, promotes flow of qi and blood and disperses heat	[29]

**Table 2:** Summary of TCM.

TCM	Type of study	Mechanisms	Reference
Weiansan	In clinic, in vivo	Anti-*Helicobacter pylori*	[17]
Wei Bi Mei granules	In vivo	Anti-*Helicobacter pylori*	[32]
Tinospora sagittata (Oliv.) Gagnep. var.craveniana (S. Y. Hu) Lo	In vivo, in vitro	Anti-*Helicobacter pylori*	[33]
Jinghua Weikang capsule	In vitro	Anti-*Helicobacter pylori*	[34]
*Atractylodes lancea* volatile oils	In vitro	Anti-*Helicobacter pylori*, anti-inflammation	[35]
*Polygonum capitatum*	In vivo,	Anti-*Helicobacter pylori*, anti-inflammation	[36]
Patchouli alcohol	In vivo, in vitro	Anti-*Helicobacter pylori*, anti-inflammation	[37]
Geniposide and genipin	In vivo, in vitro	Anti-*Helicobacter pylori*, anti-inflammation	[38]
Weiqi decoction	In vivo	Anti-inflammation, proapoptosis, antiproliferation, antiglycolytic	[39]
*Panax ginseng*	In vivo	Anti-inflammation, antioxidation	[40]
*Muscovite*	In vivo	Anti-inflammation	[41]
Xiao Tan He Wei decoction	In vivo, in vitro	Proapoptosis, antiproliferation	[42]
*Ginkgo biloba*	In vivo	Proapoptosis, antiproliferation, antioxidation	[43]
*Hericium erinaceus*	In vitro	Proapoptosis	[44]
Astragaloside IV	In vivo	Proapoptosis, antiproliferation, anti-inflammation	[45]
Weikangfu granule	In clinic	Antioxidation	[28]
Jianpiyiwei capsule	In vivo	Antioxidation	[46]
Curcumin	In vivo	Antioxidation	[47]
*Dendrobium officinale* polysaccharides	In vivo	Antioxidation	[48]
Astragaloside IV	In vivo	Antiglycolytic	[49]
Weipixiao formula	In vivo	Antiangiogenesis	[16]
Weining granules	In clinic	Antiangiogenesis	[50]
*β*-Glucan	In vivo	Antiangiogenesis, antiproliferation	[51]

## Abbreviations

GPL: Gastric precancerous lesion
TCM: Traditional Chinese medicine
GCO: Global cancer observatory
*H. pylori*: *Helicobacter pylori*
TSG: Tinospora sagittata (Oliv.) Gagnep. var. craveniana (S. Y. Hu) Lo
COX-2: Cyclo-oxygenase-2
ROS: Reactive oxygen species.
